# Structural, Hirshfeld and DFT studies of conjugated *D*–π–*A* carbazole chalcone crystal

**DOI:** 10.1107/S2056989020002054

**Published:** 2020-02-18

**Authors:** Muhamad Fikri Zaini, Ibrahim Abdul Razak, Wan Mohd Khairul, Suhana Arshad

**Affiliations:** a X-ray Crystallography Unit, School of Physics, Universiti Sains Malaysia, 11800 USM, Penang, Malaysia; b School of Fundamental Science, Universiti Malaysia Terengganu, 21030, Kuala Terengganu, Terengganu, Malaysia

**Keywords:** carbazole chalcone, crystal structure, Hirshfeld surface, DFT, mol­ecular electrostatic potential

## Abstract

A new conjugated carbazole chalcone compound, (*E*)-3-[4-(9,9a-di­hydro-8a*H*-carbazol-9-yl)phen­yl]-1-(4-nitro­phen­yl)prop-2-en-1-one (CPNC), C_27_H_18_N_2_O_3_, was synthesized using a Claisen–Schmidt condensation reaction. CPNC crystallizes in the monoclinic non-centrosymmetric space group *Cc* and adopts an *s-cis* conformation with respect to the ethyl­enic double bonds (C=O and C=C). The crystal packing features C—H⋯O and C—H⋯π inter­actions whose percentage contribution was qu­anti­fied by Hirshfeld surface analysis. Quantum chemistry calculations including geometrical optimization and mol­ecular electrostatic potential (MEP) were analysed by density functional theory (DFT) with a B3LYP/6–311 G++(d,p) basis set.

## Abstract 2

The title conjugated carbazole chalcone compound, synthesized using a Claisen–Schmidt condensation reaction, adopts an *s*-*cis* conformation with respect to the ethyl­enic double bonds (C=O and C=C).

## Chemical context   

Chalcone is a privileged structure comprising two aromatic rings that are linked by a three-carbon α,β-unsaturated carbonyl system. Chalcones demonstrate wide-ranging bio­logical activities such as anti-inflammatory and anti­cancer (Cui *et al.*, 2008 ▸; Srinivasan *et al.*, 2009 ▸; Wang *et al.*, 2013 ▸) and have applications in non-linear optics (Zaini, Arshad *et al.*, 2019 ▸). They are currently attracting considerable attention because they offer an excellent π-conjugated system within the double bond at the ethyl­enic bridge (Teo *et al.*, 2017 ▸). Furthermore, the conjugated chalcone could be enhanced with appropriate electron-pulling and electron-pushing functional groups on the benzene rings (Zhuang *et al.*, 2017 ▸). The increased involvement of donor and acceptor inter­actions in the mol­ecule improves the mol­ecular charge transfer and degree of non-linearity (Davanagere *et al.*, 2019 ▸). The high planarity and presence of stable *E* isomer in the solid state stabilizes the crystal structure (Custodio *et al.*, 2020 ▸).
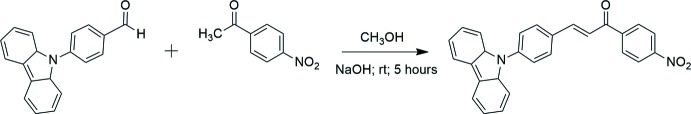


In a continuation of our studies (Zaini *et al.*, 2018 ▸; Zaini, Razak *et al.*, 2019 ▸), we report herein the synthesis and structural properties of the conjugated carbazole chalcone system of (*E*)-3-[4-(9,9a-di­hydro-8a*H*-carbazol-9-yl)phen­yl]-1-(4-nitro­phen­yl)prop-2-en-1-one (CPNC). The experimental and theoretical studies and chemical reactivity analysis are discussed.

## Structural commentary   

**Figure 1:**
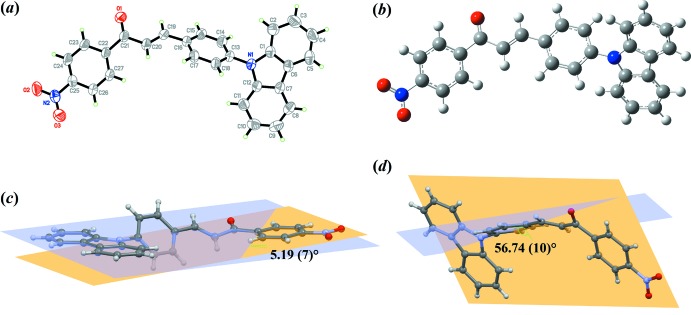
(*a*) The crystal structure of CPNC showing 50% probability ellipsoids, (*b*) the optimized structure, (*c*) the dihedral angle between the nitro­benzene plane and the 9*H*-carbazole unit and (*d*) the dihedral angle between the nitro­benzene plane and the phenyl ring of the 9-phenyl­carbazole unit.

CPNC is composed of 9-phenyl­carbazole and nitro­benzene moieties, which represent donor and acceptor groups, connected by an ethyl­enic bridge. The mol­ecular and optimized structures of the CPNC with assigned atom-numbering scheme are illustrated in Fig. 1 ▸. The geometrical optimization of CPNC was computed with the *Gaussian09W* software package (Frisch *et al.*, 2009 ▸) using the DFT method and the B3LYP/6-311G++(d,p) basis set without enforcing any mol­ecular symmetry constraints. There is good agreement between the experimental and optimized structures (see the table in the supporting information), indicating that the basis set used was appropriate in both isolated conditions and the solid-state phase.

CPNC crystallizes in the monoclinic *Cc* space group with four mol­ecules per unit cell. Its mol­ecular structure exhibits an *s*-*cis* configuration with respect to the ethyl­enic bridge consisting of carbonyl (C=O; 1.215 (3) Å (experimental), 1.223 Å (DFT)] and carbon–carbon double bond (C=C; 1.320 (3) Å (experimental), 1.348 Å (DFT)]. The CPNC mol­ecule is twisted slightly at the C21—C22 bond, with a C20—C21—C22—C27 torsion angle of −10.4 (3)° (DFT value = −21.3°). The experimental and theoretical C15—C16—C19—C20 torsion angles are 158.6 (3) and 178.8°, respectively. The 9-phenyl­carbazole C13—N1 bond is also observed to be twisted [C1—N1—C13—C14 51.8 (4)° (in experimental) and 53.2° (DFT). The small discrepancies in the torsion angles between the experimental and calculated DFT results are caused by the involvement of inter­molecular inter­actions, which are negligible during the optimization process (Arshad *et al.*, 2018 ▸).

There is also a twist [dihedral angle = 25.30 (17)°] between the mean planes of the nitro­phenyl group [N2/O2/O3/C22–C27; maximum deviation of 0.023 (2) Å at atom O3] and the enone unit [O1/C19–C21; maximum deviation of 0.109 (2) Å at atom C21]. Meanwhile, the enone bridge forms dihedral angles of 31.52 (18) and 21.77 (16)°, respectively, with the C13–C18 phenyl ring and the 9*H*-carbazole unit [N1/C1–C12; maximum deviation of 0.041 (3) Å at atom C2].

The 9*H*-carbazole unit and the C13–C18 phenyl ring subtend a dihedral angle of 53.26 (10)°, which is similar to the dihedral angle of 53.8 (3) between the bridge aromatic ring and the 9*H*-carbazole unit in the related compound 2-[4-(9*H-*carbazol-9-yl)benzyl­idene]-2,3-di­hydro­inden-1-one (Kim *et al.*, 2011 ▸). The 9*H*-carbazole moiety is nearly co-planar with the nitro­benzene unit, making a dihedral angle of 5.19 (7)° (Fig. 1 ▸ *c*). This planar nature is possibly due to steric repulsion by the hydrogen atoms of the aromatic rings, leading to a small π-electron delocalization. However, the phenyl ring of the 9-phenyl­carbazole moiety subtends a large dihedral angle to the nitro­benzene group of 56.74 (10)° (Fig. 1 ▸ *d*), which tends to suppress the extension of the conjugation effect through the enone moiety.

## Supra­molecular features   

**Figure 2:**
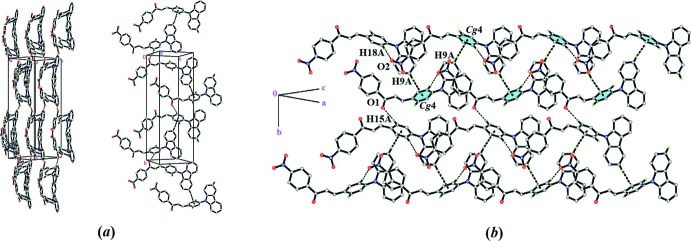
The packing of CPNC showing (*a*) C—H⋯O and C—H⋯π inter­actions (dashed lines) and (*b*) C—H⋯π inter­actions forming an infinite chain along the *ac*-plane direction.

The crystal structure of CPNC is built up in a cluster pattern style where the mol­ecules are linked to each other along the *b*-axis direction *via* C15—H15*A*⋯O1 inter­actions (Table 1 ▸), as shown in Fig. 2 ▸ *a*. The tilted distortion of 9-phenyl­carbazole ring system is the results of the C18—H18*A*⋯O2 inter­action involving the nitro group, which links the mol­ecules in a head-to-tail arrangement, propagating diagonally along the *ac* direction. Weak C9—H9*A*⋯*Cg*4 inter­actions involving the C13-C18 phenyl ring and a carbazole hydrogen of carbazole moiety link the mol­ecules into infinite chains, as depicted in Fig. 2 ▸ *b*. Overall, the inter­molecular C—H⋯O and C—H⋯π inter­actions of CPNC generate a three-dimensional network.

## Hirshfeld surface analysis   

**Figure 3:**
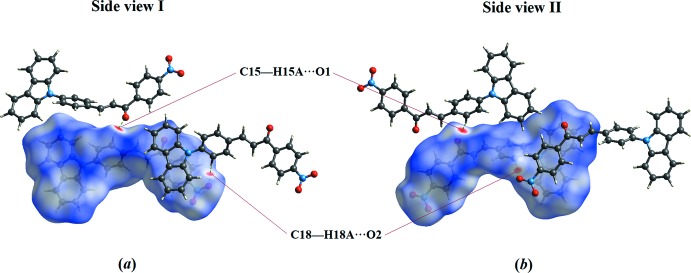
The *d*_norm_ surfaces showing the inter­molecular inter­actions in CPNC: (*a*) front and (*b*) back.

**Figure 4:**
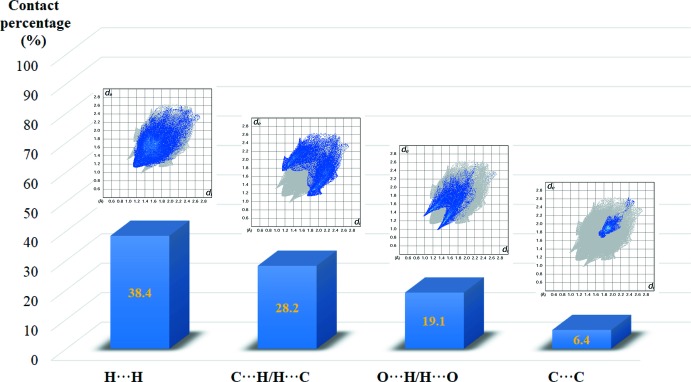
Qu­anti­fication of different types of contacts and respective fingerprints plots.

**Figure 5:**
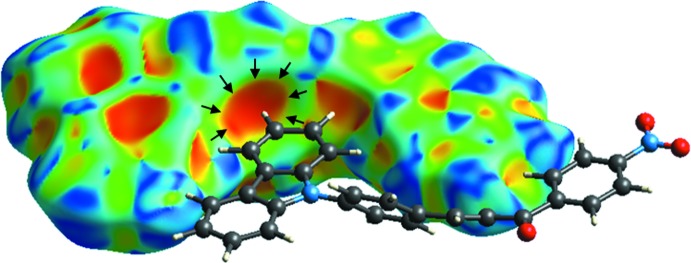
Representation of the C—H⋯π inter­actions (indicated by black arrows).

Hirshfeld surface analysis is used to gain a clear understanding of the mol­ecular structure inter­action and visualize them graphically. The Hirshfeld surface and related two-dimensional fingerprint plots were generated using *Crystal Explorer3.1* (Wolff *et al.*, 2012 ▸). In the *d*_norm_ surface (Fig. 3 ▸), the bright-red spots indicate the involvement of inter­molecular C—H⋯O inter­actions. The fingerprint plots (Ternavisk *et al.*, 2014 ▸) (Fig. 4 ▸) indicate the percentage contribution of the H⋯H, C⋯H/H⋯C, O⋯H/H⋯O and C⋯C contacts. The H⋯H contacts make the largest contribution to the Hirshfeld surface (38.4%) followed by the C⋯H/H⋯C contacts (28.2%), which are represented as a pair of characteristic wings. The O⋯H/H⋯O (19.1%) contacts display two symmetrical narrow spikes, which confirm the existence of C—H⋯O inter­actions. In addition, the presence of weak inter­molecular C—H⋯π inter­actions can be seen as an orange region marked with black arrows in the shape-index surface (Fig. 5 ▸).

## Mol­ecular electrostatic potential (MEP) analysis   

**Figure 6:**
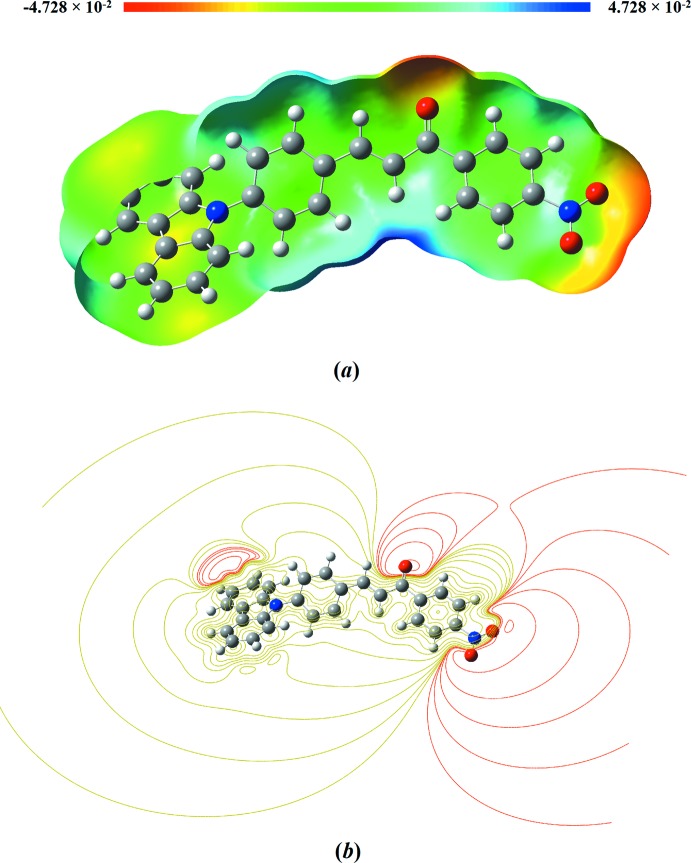
(*a*) Mol­ecular electrostatic potentials (MEP) and (*b*) its contour map mapped on the electron density surface calculated by using the DFT/B3LYP/6–311 G++(d,p) basis set.

The reactive sites of a mol­ecule can be investigated using mol­ecular electrostatic potential (MEP) analysis (Barakat *et al.*, 2015 ▸). In this study, DFT with the B3LYP/6-311G++(d,p) basis set was utilized to predict the possible location of the nucleophilic and electrophilic attacks. The MEP surface with a colour code from red (−0.04728 a.u) to blue (0.04728 a.u) is depicted in Fig. 6 ▸ *a*. The carbonyl and nitro groups are nucleophilic (electron-rich) sites in the red-coloured region, while the blue colour indicates the electrophilic (electron-deficient) site localized on the hydrogen atom. These reactive sites are responsible for inter­molecular inter­actions where the red and blue spots suggest the strongest repulsion site (electrophilic attack) and strongest attraction site (nucleophilic attack), respectively. The MEP results are further supported by the electrostatic potential contour map showing the iso-surface lines shown in Fig. 6 ▸ *b* where the red lines refer to the strong electron-withdrawing atoms such as in carbonyl and nitro substituents.

## Database survey   

A search of the Cambridge Structural Database (CSD, Version 5.40, last update February 2019; Groom *et al.*, 2016 ▸) revealed one closely related 9-phenyl­carbazole chalcone, namely 1-(anthracen-9-yl)-3-[4-(9*H*-carbazol-9-yl)phen­yl]prop-2-en-1-one (refcode ZIJPUG; Zainuri *et al.*, 2018 ▸) with an anthracene system as the ketone substituent. Another similar compound is 2-[4-(9*H*-carbazol-9-yl)benzyl­idene]indan-1-one (ISADOW; Kim *et al.*, 2011 ▸) in which the 9-phenyl­carbazole unit is attached to a 2,3-di­hydro-1*H*-inden-1-one moiety. The two crystals were grown by different methods, ZIJPUG by slow evaporation from acetone solution and ISADOW by solvent diffusion using di­chloro­methane and hexane. The reported mol­ecular structures of ZIJPUG and ISADOW exhibit a π-bridge linker of an enone moiety and the aromatic ring of 9-phenyl­carbazole, respectively. Furthermore, the C16—C17—C18—C19 torsion angle in ZIJPUG [−16.4 (3)°] indicates a slight twist, which is which comparable to that in ISADOW [C8—C10—C11—C12 = 178.6 (2)°].

## Synthesis and crystallization   

4′-Nitro­aceto­phenone (5 mmol) and *N*-(4-formyl­phen­yl)carbazole (5 mmol) were dissolved in 20 mL of methanol and then a catalytic amount of sodium hydroxide solution (5 mL, 20%) was added dropwise under continuous stirring for about 5–6 h at room temperature until a precipitate formed. This was filtered off, washed successively with distilled water and recrystallized from acetone solution, yielding orange block-shaped crystals suitable for X-ray diffraction analysis.

## Refinement   

Crystal data, data collection and structure refinement details are summarized in Table 2 ▸. All H atoms were positioned geometrically (C—H = 0.93 Å) and refined using a riding model with *U*_iso_(H) = 1.2*U*_eq_(C).

## Supplementary Material

Crystal structure: contains datablock(s) I. DOI: 10.1107/S2056989020002054/ex2028sup1.cif

Structure factors: contains datablock(s) I. DOI: 10.1107/S2056989020002054/ex2028Isup2.hkl

Supporting information file. DOI: 10.1107/S2056989020002054/ex2028Isup4.cml

SI Comparison between the calculated optimized and X-ray geometrical parameters for the CPNC compound. DOI: 10.1107/S2056989020002054/ex2028sup3.docx

CCDC reference: 1967669

Additional supporting information:  crystallographic information; 3D view; checkCIF report

## Figures and Tables

**Table 1:** Hydrogen-bond geometry (Å, °) *Cg*4 is the centroid of the C13–C18 ring.

*D*—H⋯*A*	*D*—H	H⋯*A*	*D*⋯*A*	*D*—H⋯*A*
C15—H15*A*⋯O1^i^	0.93	2.42	3.291 (3)	155
C18—H18*A*⋯O2^ii^	0.93	2.56	3.490 (3)	173
C9—H9*A*⋯*Cg*4^iii^	0.93	2.89	3.758 (3)	155

Symmetry codes: (i) 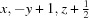; (ii) 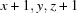; (iii) 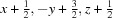.

**Table 2:** Experimental details

Crystal data	
Chemical formula	C_27_H_18_N_2_O_3_
*M*_r_	418.43
Crystal system, space group	Monoclinic, *C* *c*
Temperature (K)	293
*a*, *b*, *c* (Å)	9.9690 (5), 24.8828 (15), 8.3049 (4)
β (°)	94.356 (1)
*V* (Å^3^)	2054.13 (19)
*Z*	4
Radiation type	Mo *K*α
μ (mm^−1^)	0.09
Crystal size (mm)	0.54 × 0.38 × 0.23

Data collection	
Diffractometer	Bruker APEXII CCD
Absorption correction	Multi-scan (*SADABS*; Bruker 2015 ▸)
*T*_min_, *T*_max_	0.783, 0.942
No. of measured, independent and observed [*I* > 2σ(*I*)] reflections	39335, 5995, 5046
*R*_int_	0.033
(sin θ/λ)_max_ (Å^−1^)	0.704

Refinement	
*R*[*F* ^2^ > 2σ(*F* ^2^)], *wR*(*F* ^2^), *S*	0.041, 0.114, 1.04
No. of reflections	5995
No. of parameters	289
No. of restraints	2
H-atom treatment	H-atom parameters constrained
Δρ_max_, Δρ_min_ (e Å^−3^)	0.19, −0.15
Absolute structure	Flack *x* determined using 2241 quotients [(*I* ^+^)−(*I* ^−^)]/[(*I* ^+^)+(*I* ^−^)] (Parsons et al., 2013 ▸)
Absolute structure parameter	−0.1 (3)

Computer programs: *APEX2* and *SAINT* (Bruker 2015 ▸), *SHELXS97* and *SHELXTL* (Sheldrick, 2008 ▸), *SHELXL2014* (Sheldrick, 2015 ▸) and *Mercury* (Macrae *et al.*, 2020 ▸).

## supplementary crystallographic information

### Crystal data

**Table:**

C_27_H_18_N_2_O_3_	*F*(000) = 872
*M**_r_* = 418.43	*D*_x_ = 1.353 Mg m^−^^3^
Monoclinic, *C**c*	Mo *K*α radiation, λ = 0.71073 Å
*a* = 9.9690 (5) Å	Cell parameters from 9847 reflections
*b* = 24.8828 (15) Å	θ = 2.2–29.5°
*c* = 8.3049 (4) Å	µ = 0.09 mm^−^^1^
β = 94.356 (1)°	*T* = 293 K
*V* = 2054.13 (19) Å^3^	Block, orange
*Z* = 4	0.54 × 0.38 × 0.23 mm

### Data collection

**Table:**

Bruker APEXII CCD diffractometer	5046 reflections with *I* > 2σ(*I*)
φ and ω scans	*R*_int_ = 0.033
Absorption correction: multi-scan (SADABS; Bruker 2015)	θ_max_ = 30.0°, θ_min_ = 1.6°
*T*_min_ = 0.783, *T*_max_ = 0.942	*h* = −13→14
39335 measured reflections	*k* = −34→34
5995 independent reflections	*l* = −11→11

### Refinement

**Table:**

Refinement on *F*^2^	Hydrogen site location: inferred from neighbouring sites
Least-squares matrix: full	H-atom parameters constrained
*R*[*F*^2^ > 2σ(*F*^2^)] = 0.041	*w* = 1/[σ^2^(*F*_o_^2^) + (0.0576*P*)^2^ + 0.455*P*] where *P* = (*F*_o_^2^ + 2*F*_c_^2^)/3
*wR*(*F*^2^) = 0.114	(Δ/σ)_max_ < 0.001
*S* = 1.03	Δρ_max_ = 0.19 e Å^−^^3^
5995 reflections	Δρ_min_ = −0.15 e Å^−^^3^
289 parameters	Absolute structure: Flack *x* determined using 2241 quotients [(*I*^+^)-(*I*^-^)]/[(*I*^+^)+(*I*^-^)] (Parsons et al., 2013)
2 restraints	Absolute structure parameter: −0.1 (3)

### Special details

**Table:**

Experimental. The following wavelength and cell were deduced by SADABS from the direction cosines etc. They are given here for emergency use only: CELL 0.71074 8.313 9.985 13.418 68.212 88.424 85.648
Geometry. All esds (except the esd in the dihedral angle between two l.s. planes) are estimated using the full covariance matrix. The cell esds are taken into account individually in the estimation of esds in distances, angles and torsion angles; correlations between esds in cell parameters are only used when they are defined by crystal symmetry. An approximate (isotropic) treatment of cell esds is used for estimating esds involving l.s. planes.

### Fractional atomic coordinates and isotropic or equivalent isotropic displacement parameters (Å^2^)

**Table:**

	*x*	*y*	*z*	*U*_iso_*/*U*_eq_	
O1	0.2822 (2)	0.52900 (7)	0.1628 (3)	0.0715 (6)	
O2	0.0014 (2)	0.71634 (9)	−0.3674 (2)	0.0685 (5)	
O3	0.1029 (2)	0.77955 (9)	−0.2279 (3)	0.0681 (5)	
N1	0.7901 (2)	0.62943 (8)	0.9806 (2)	0.0491 (4)	
N2	0.0790 (2)	0.73213 (9)	−0.2556 (2)	0.0514 (5)	
C1	0.8488 (2)	0.59669 (10)	1.1043 (3)	0.0494 (5)	
C2	0.8433 (3)	0.54134 (11)	1.1238 (3)	0.0640 (7)	
H2A	0.7917	0.5198	1.0514	0.077*	
C3	0.9176 (4)	0.51939 (13)	1.2555 (4)	0.0768 (9)	
H3A	0.9161	0.4824	1.2707	0.092*	
C4	0.9943 (4)	0.55101 (14)	1.3656 (4)	0.0748 (9)	
H4A	1.0435	0.5349	1.4522	0.090*	
C5	0.9978 (3)	0.60597 (13)	1.3474 (3)	0.0633 (7)	
H5A	1.0484	0.6272	1.4215	0.076*	
C6	0.9240 (2)	0.62937 (10)	1.2159 (3)	0.0482 (5)	
C7	0.9091 (2)	0.68404 (10)	1.1603 (3)	0.0462 (5)	
C8	0.9545 (3)	0.73328 (11)	1.2227 (3)	0.0574 (6)	
H8A	1.0090	0.7348	1.3185	0.069*	
C9	0.9177 (3)	0.77957 (11)	1.1410 (4)	0.0644 (7)	
H9A	0.9483	0.8126	1.1815	0.077*	
C10	0.8351 (3)	0.77768 (11)	0.9983 (4)	0.0615 (6)	
H10A	0.8106	0.8096	0.9459	0.074*	
C11	0.7885 (3)	0.72941 (10)	0.9322 (3)	0.0538 (6)	
H11A	0.7340	0.7284	0.8362	0.065*	
C12	0.8262 (2)	0.68269 (9)	1.0148 (3)	0.0447 (4)	
C13	0.7077 (2)	0.61190 (9)	0.8430 (3)	0.0467 (5)	
C14	0.5978 (3)	0.57950 (11)	0.8617 (3)	0.0565 (6)	
H14A	0.5780	0.5685	0.9643	0.068*	
C15	0.5166 (3)	0.56331 (11)	0.7274 (3)	0.0558 (6)	
H15A	0.4435	0.5410	0.7407	0.067*	
C16	0.5429 (2)	0.58002 (9)	0.5731 (3)	0.0481 (5)	
C17	0.6564 (2)	0.61148 (10)	0.5552 (3)	0.0511 (5)	
H17A	0.6774	0.6219	0.4525	0.061*	
C18	0.7384 (2)	0.62741 (10)	0.6890 (3)	0.0503 (5)	
H18A	0.8139	0.6485	0.6758	0.060*	
C19	0.4473 (3)	0.56599 (10)	0.4358 (3)	0.0528 (5)	
H19A	0.3907	0.5369	0.4499	0.063*	
C20	0.4339 (3)	0.59058 (10)	0.2947 (3)	0.0538 (6)	
H20A	0.4922	0.6184	0.2730	0.065*	
C21	0.3268 (2)	0.57442 (10)	0.1701 (3)	0.0511 (5)	
C22	0.2696 (2)	0.61668 (10)	0.0544 (3)	0.0458 (5)	
C23	0.1803 (3)	0.60042 (11)	−0.0741 (3)	0.0568 (6)	
H23A	0.1623	0.5641	−0.0904	0.068*	
C24	0.1188 (2)	0.63807 (11)	−0.1771 (3)	0.0550 (6)	
H24A	0.0601	0.6275	−0.2637	0.066*	
C25	0.1462 (2)	0.69147 (10)	−0.1489 (3)	0.0452 (5)	
C26	0.2350 (2)	0.70923 (10)	−0.0246 (3)	0.0510 (5)	
H26A	0.2522	0.7457	−0.0089	0.061*	
C27	0.2975 (3)	0.67082 (10)	0.0758 (3)	0.0518 (5)	
H27A	0.3593	0.6816	0.1590	0.062*	

### Atomic displacement parameters (Å^2^)

**Table:**

	*U*^11^	*U*^22^	*U*^33^	*U*^12^	*U*^13^	*U*^23^
O1	0.0810 (13)	0.0456 (9)	0.0816 (13)	0.0062 (9)	−0.0343 (10)	−0.0086 (9)
O2	0.0650 (11)	0.0839 (14)	0.0528 (10)	0.0021 (10)	−0.0193 (8)	0.0097 (9)
O3	0.0688 (12)	0.0606 (11)	0.0728 (12)	0.0061 (9)	−0.0075 (9)	0.0078 (9)
N1	0.0542 (11)	0.0498 (10)	0.0410 (9)	−0.0063 (8)	−0.0105 (8)	−0.0006 (8)
N2	0.0444 (10)	0.0643 (13)	0.0449 (9)	0.0046 (9)	−0.0001 (8)	0.0055 (8)
C1	0.0538 (13)	0.0540 (12)	0.0393 (10)	−0.0010 (10)	−0.0041 (9)	0.0003 (9)
C2	0.0829 (19)	0.0556 (14)	0.0521 (14)	−0.0008 (13)	−0.0045 (13)	0.0000 (11)
C3	0.110 (3)	0.0587 (16)	0.0597 (16)	0.0113 (16)	−0.0051 (16)	0.0112 (13)
C4	0.093 (2)	0.0776 (19)	0.0507 (14)	0.0152 (17)	−0.0109 (14)	0.0122 (13)
C5	0.0670 (16)	0.0815 (19)	0.0394 (11)	0.0020 (14)	−0.0092 (10)	−0.0001 (12)
C6	0.0484 (11)	0.0582 (13)	0.0375 (10)	−0.0017 (10)	−0.0005 (8)	−0.0012 (9)
C7	0.0419 (10)	0.0578 (13)	0.0386 (10)	−0.0064 (9)	0.0017 (8)	−0.0025 (9)
C8	0.0582 (14)	0.0679 (15)	0.0461 (12)	−0.0185 (12)	0.0054 (10)	−0.0105 (11)
C9	0.0772 (18)	0.0560 (15)	0.0621 (16)	−0.0218 (13)	0.0188 (14)	−0.0103 (12)
C10	0.0718 (17)	0.0531 (13)	0.0609 (15)	−0.0049 (12)	0.0137 (13)	0.0060 (11)
C11	0.0555 (13)	0.0565 (14)	0.0491 (12)	0.0000 (10)	0.0014 (10)	0.0046 (10)
C12	0.0421 (10)	0.0514 (12)	0.0401 (10)	−0.0052 (8)	−0.0003 (8)	−0.0017 (8)
C13	0.0477 (11)	0.0486 (11)	0.0417 (10)	−0.0030 (9)	−0.0096 (8)	−0.0028 (9)
C14	0.0618 (14)	0.0628 (15)	0.0436 (11)	−0.0136 (11)	−0.0051 (10)	0.0017 (10)
C15	0.0591 (14)	0.0527 (13)	0.0536 (12)	−0.0166 (11)	−0.0089 (10)	0.0029 (10)
C16	0.0539 (12)	0.0419 (10)	0.0459 (10)	0.0017 (9)	−0.0129 (9)	−0.0031 (8)
C17	0.0518 (12)	0.0587 (13)	0.0413 (10)	0.0008 (10)	−0.0064 (9)	0.0017 (9)
C18	0.0454 (11)	0.0587 (13)	0.0454 (11)	−0.0056 (10)	−0.0070 (9)	0.0029 (9)
C19	0.0589 (13)	0.0424 (11)	0.0541 (13)	−0.0001 (9)	−0.0154 (11)	−0.0054 (9)
C20	0.0541 (13)	0.0522 (13)	0.0518 (12)	0.0028 (10)	−0.0176 (10)	−0.0065 (10)
C21	0.0525 (12)	0.0490 (12)	0.0490 (12)	0.0100 (9)	−0.0150 (9)	−0.0099 (9)
C22	0.0441 (11)	0.0494 (11)	0.0419 (10)	0.0043 (9)	−0.0099 (8)	−0.0067 (8)
C23	0.0559 (13)	0.0528 (13)	0.0579 (13)	0.0003 (10)	−0.0212 (11)	−0.0102 (10)
C24	0.0516 (13)	0.0597 (14)	0.0504 (12)	0.0011 (10)	−0.0188 (10)	−0.0065 (10)
C25	0.0398 (10)	0.0572 (12)	0.0380 (9)	0.0029 (9)	−0.0022 (8)	0.0008 (9)
C26	0.0561 (13)	0.0532 (12)	0.0419 (11)	−0.0033 (10)	−0.0086 (9)	−0.0021 (9)
C27	0.0566 (13)	0.0550 (13)	0.0412 (10)	−0.0018 (10)	−0.0131 (9)	−0.0071 (9)

### Geometric parameters (Å, º)

**Table:**

O1—C21	1.215 (3)	C11—H11A	0.9300
O2—N2	1.227 (3)	C13—C14	1.378 (4)
O3—N2	1.222 (3)	C13—C18	1.393 (3)
N1—C12	1.397 (3)	C14—C15	1.387 (3)
N1—C1	1.404 (3)	C14—H14A	0.9300
N1—C13	1.425 (3)	C15—C16	1.391 (3)
N2—C25	1.472 (3)	C15—H15A	0.9300
C1—C2	1.388 (4)	C16—C17	1.394 (4)
C1—C6	1.406 (3)	C16—C19	1.471 (3)
C2—C3	1.386 (4)	C17—C18	1.386 (3)
C2—H2A	0.9300	C17—H17A	0.9300
C3—C4	1.390 (5)	C18—H18A	0.9300
C3—H3A	0.9300	C19—C20	1.320 (3)
C4—C5	1.377 (5)	C19—H19A	0.9300
C4—H4A	0.9300	C20—C21	1.485 (3)
C5—C6	1.396 (3)	C20—H20A	0.9300
C5—H5A	0.9300	C21—C22	1.507 (3)
C6—C7	1.441 (3)	C22—C27	1.384 (3)
C7—C8	1.392 (3)	C22—C23	1.397 (3)
C7—C12	1.412 (3)	C23—C24	1.381 (3)
C8—C9	1.373 (4)	C23—H23A	0.9300
C8—H8A	0.9300	C24—C25	1.373 (4)
C9—C10	1.392 (4)	C24—H24A	0.9300
C9—H9A	0.9300	C25—C26	1.380 (3)
C10—C11	1.386 (4)	C26—C27	1.385 (3)
C10—H10A	0.9300	C26—H26A	0.9300
C11—C12	1.387 (3)	C27—H27A	0.9300
			
C12—N1—C1	108.39 (18)	C18—C13—N1	119.9 (2)
C12—N1—C13	125.30 (19)	C13—C14—C15	120.0 (2)
C1—N1—C13	126.31 (19)	C13—C14—H14A	120.0
O3—N2—O2	123.6 (2)	C15—C14—H14A	120.0
O3—N2—C25	118.5 (2)	C14—C15—C16	121.0 (2)
O2—N2—C25	117.9 (2)	C14—C15—H15A	119.5
C2—C1—N1	130.1 (2)	C16—C15—H15A	119.5
C2—C1—C6	121.4 (2)	C15—C16—C17	118.5 (2)
N1—C1—C6	108.6 (2)	C15—C16—C19	119.1 (2)
C3—C2—C1	117.4 (3)	C17—C16—C19	122.3 (2)
C3—C2—H2A	121.3	C18—C17—C16	120.6 (2)
C1—C2—H2A	121.3	C18—C17—H17A	119.7
C2—C3—C4	122.0 (3)	C16—C17—H17A	119.7
C2—C3—H3A	119.0	C17—C18—C13	120.0 (2)
C4—C3—H3A	119.0	C17—C18—H18A	120.0
C5—C4—C3	120.5 (3)	C13—C18—H18A	120.0
C5—C4—H4A	119.8	C20—C19—C16	126.4 (2)
C3—C4—H4A	119.8	C20—C19—H19A	116.8
C4—C5—C6	118.9 (3)	C16—C19—H19A	116.8
C4—C5—H5A	120.5	C19—C20—C21	120.8 (2)
C6—C5—H5A	120.5	C19—C20—H20A	119.6
C5—C6—C1	119.8 (2)	C21—C20—H20A	119.6
C5—C6—C7	132.8 (2)	O1—C21—C20	121.9 (2)
C1—C6—C7	107.34 (19)	O1—C21—C22	119.9 (2)
C8—C7—C12	119.5 (2)	C20—C21—C22	118.2 (2)
C8—C7—C6	133.6 (2)	C27—C22—C23	119.3 (2)
C12—C7—C6	106.9 (2)	C27—C22—C21	122.34 (19)
C9—C8—C7	119.2 (2)	C23—C22—C21	118.3 (2)
C9—C8—H8A	120.4	C24—C23—C22	120.3 (2)
C7—C8—H8A	120.4	C24—C23—H23A	119.9
C8—C9—C10	120.7 (2)	C22—C23—H23A	119.9
C8—C9—H9A	119.6	C25—C24—C23	118.5 (2)
C10—C9—H9A	119.6	C25—C24—H24A	120.7
C11—C10—C9	121.7 (3)	C23—C24—H24A	120.7
C11—C10—H10A	119.2	C24—C25—C26	123.1 (2)
C9—C10—H10A	119.2	C24—C25—N2	119.1 (2)
C10—C11—C12	117.4 (2)	C26—C25—N2	117.8 (2)
C10—C11—H11A	121.3	C25—C26—C27	117.6 (2)
C12—C11—H11A	121.3	C25—C26—H26A	121.2
C11—C12—N1	129.7 (2)	C27—C26—H26A	121.2
C11—C12—C7	121.5 (2)	C22—C27—C26	121.2 (2)
N1—C12—C7	108.8 (2)	C22—C27—H27A	119.4
C14—C13—C18	119.8 (2)	C26—C27—H27A	119.4
C14—C13—N1	120.3 (2)		
			
C12—N1—C1—C2	−179.4 (3)	C12—N1—C13—C18	52.0 (3)
C13—N1—C1—C2	0.6 (4)	C1—N1—C13—C18	−128.0 (3)
C12—N1—C1—C6	−0.9 (3)	C18—C13—C14—C15	−1.3 (4)
C13—N1—C1—C6	179.1 (2)	N1—C13—C14—C15	178.9 (2)
N1—C1—C2—C3	176.8 (3)	C13—C14—C15—C16	−1.1 (4)
C6—C1—C2—C3	−1.6 (4)	C14—C15—C16—C17	2.9 (4)
C1—C2—C3—C4	0.5 (5)	C14—C15—C16—C19	−174.4 (2)
C2—C3—C4—C5	0.5 (6)	C15—C16—C17—C18	−2.4 (4)
C3—C4—C5—C6	−0.5 (5)	C19—C16—C17—C18	174.9 (2)
C4—C5—C6—C1	−0.5 (4)	C16—C17—C18—C13	0.1 (4)
C4—C5—C6—C7	−178.0 (3)	C14—C13—C18—C17	1.8 (4)
C2—C1—C6—C5	1.5 (4)	N1—C13—C18—C17	−178.4 (2)
N1—C1—C6—C5	−177.1 (2)	C15—C16—C19—C20	158.6 (3)
C2—C1—C6—C7	179.7 (2)	C17—C16—C19—C20	−18.7 (4)
N1—C1—C6—C7	1.1 (3)	C16—C19—C20—C21	−175.9 (2)
C5—C6—C7—C8	−5.4 (5)	C19—C20—C21—O1	−27.7 (4)
C1—C6—C7—C8	176.8 (3)	C19—C20—C21—C22	150.0 (2)
C5—C6—C7—C12	177.0 (3)	O1—C21—C22—C27	167.4 (3)
C1—C6—C7—C12	−0.8 (2)	C20—C21—C22—C27	−10.4 (3)
C12—C7—C8—C9	−0.1 (3)	O1—C21—C22—C23	−9.5 (4)
C6—C7—C8—C9	−177.5 (3)	C20—C21—C22—C23	172.7 (2)
C7—C8—C9—C10	0.6 (4)	C27—C22—C23—C24	−1.1 (4)
C8—C9—C10—C11	−0.8 (4)	C21—C22—C23—C24	175.9 (2)
C9—C10—C11—C12	0.6 (4)	C22—C23—C24—C25	−0.8 (4)
C10—C11—C12—N1	177.0 (2)	C23—C24—C25—C26	1.7 (4)
C10—C11—C12—C7	−0.1 (3)	C23—C24—C25—N2	−178.7 (2)
C1—N1—C12—C11	−177.0 (2)	O3—N2—C25—C24	179.1 (2)
C13—N1—C12—C11	3.0 (4)	O2—N2—C25—C24	−0.6 (3)
C1—N1—C12—C7	0.4 (2)	O3—N2—C25—C26	−1.3 (3)
C13—N1—C12—C7	−179.6 (2)	O2—N2—C25—C26	179.0 (2)
C8—C7—C12—C11	−0.1 (3)	C24—C25—C26—C27	−0.6 (4)
C6—C7—C12—C11	177.9 (2)	N2—C25—C26—C27	179.7 (2)
C8—C7—C12—N1	−177.8 (2)	C23—C22—C27—C26	2.3 (4)
C6—C7—C12—N1	0.2 (2)	C21—C22—C27—C26	−174.6 (2)
C12—N1—C13—C14	−128.2 (3)	C25—C26—C27—C22	−1.4 (4)
C1—N1—C13—C14	51.8 (4)		

### Hydrogen-bond geometry (Å, º)

*Cg*4 is the centroid of the C13–C18 ring.

**Table:**

*D*—H···*A*	*D*—H	H···*A*	*D*···*A*	*D*—H···*A*
C15—H15*A*···O1^i^	0.93	2.42	3.291 (3)	155
C18—H18*A*···O2^ii^	0.93	2.56	3.490 (3)	173
C9—H9*A*···*Cg*4^iii^	0.93	2.89	3.758 (3)	155

Symmetry codes: (i) *x*, −*y*+1, *z*+1/2; (ii) *x*+1, *y*, *z*+1; (iii) *x*+1/2, −*y*+3/2, *z*+1/2.

## References

[bb1] Arshad, S., Zainuri, D. A., Khalib, N. C., Thanigaimani, K., Rosli, M. M., Razak, I. A., Sulaiman, S. F., Hashim, N. S. & Ooi, K. L. (2018). *Mol. Cryst. Liq. Cryst.* **664**, 218–240.

[bb2] Barakat, A., Al-Majid, A. M., Soliman, S. M., Mabkhot, Y. N., Ali, M., Ghabbour, H. A., Fun, H.-K. & Wadood, A. (2015). *Chem. Cent. J.* **9**, 35. 10.1186/s13065-015-0112-5 PMC4477317 26106444

[bb19] Bruker (2015). *APEX2*, *SAINT* and *SADABS*. Bruker AXS Inc., Madison, Wisconsin, USA.

[bb3] Cui, Y., Ao, M., Hu, J. & Yu, L. (2008). *Z. Naturforsch. C.* **63**, 361–365. 10.1515/znc-2008-5-609 18669021

[bb4] Custodio, J. M. F., Guimarães-Neto, J. J. A., Awad, R., Queiroz, J. E., Verde, G. M. V., Mottin, M., Neves, B. J., Andrade, C. H., Aquino, G. L. B., Valverde, C., Osório, F. A. P., Baseia, B. & Napolitano, H. B. (2020). *Arab. J. Chem*. 13, 3362–3371.

[bb7] Davanagere, H., Jayarama, A., Patil, P. S. G., Maidur, S. R., Quah, C. K. & Kwong, H. C. (2019). *Appl. Phys. A*, **125**, article No.309.

[bb5] Frisch, M. J., Trucks, G. W., Schlegel, H. B., Scuseria, G. E., Robb, M. A., Cheeseman, J. R., Scalmani, G., Barone, V., Mennucci, B., Petersson, G. A., Nakatsuji, H., Caricato, M., Li, X., Hratchian, H. P., Izmaylov, A. F., Bloino, J., Zheng, G., Sonnenberg, J. L., Hada, M., Ehara, M., Toyota, K., Fukuda, R., Hasegawa, J., Ishida, M., Nakajima, T., Honda, Y., Kitao, O., Nakai, H. & Vreven, T. (2009). *Gaussian 09, Revision B. 01*. Gaussian, Inc., Wallingford, CT, USA.

[bb6] Groom, C. R., Bruno, I. J., Lightfoot, M. P. & Ward, S. C. (2016). *Acta Cryst.* B**72**, 171–179. 10.1107/S2052520616003954 PMC4822653 27048719

[bb8] Kim, B.-S., Kim, S.-H., Matsumoto, S. & Son, Y.-A. (2011). *Z. Krist. New Cryst. Struct.* **226**, 177–178.

[bb23] Macrae, C. F., Sovago, I., Cottrell, S. J., Galek, P. T. A., McCabe, P., Pidcock, E., Platings, M., Shields, G. P., Stevens, J. S., Towler, M. & Wood, P. A. (2020). *J. Appl. Cryst.* **53**, 226–235. 10.1107/S1600576719014092 PMC6998782 32047413

[bb22] Parsons, S., Flack, H. D. & Wagner, T. (2013). *Acta Cryst.* B**69**, 249–259. 10.1107/S2052519213010014 PMC3661305 23719469

[bb20] Sheldrick, G. M. (2008). *Acta Cryst* A**64**, 112–122. 10.1107/S0108767307043930 18156677

[bb21] Sheldrick, G. M. (2015). *Acta Cryst* C**71**, 3–8.

[bb9] Srinivasan, B., Johnson, T. E., Lad, R. & Xing, C. (2009). *J. Med. Chem.* **52**, 7228–7235. 10.1021/jm901278z 19883086

[bb10] Teo, K. Y., Tiong, M. H., Wee, H. Y., Jasin, N., Liu, Z.-Q., Shiu, M. Y., Tang, J. Y., Tsai, J.-K., Rahamathullah, R., Khairul, W. M. & Tay, M. G. (2017). *J. Mol. Struct.* **1143**, 42–48.

[bb11] Ternavisk, R. R., Camargo, A. J., Machado, F. B., Rocco, J. A., Aquino, G. L., Silva, V. H. & Napolitano, H. B. (2014). *J. Mol. Model.* **20**, 2526. 10.1007/s00894-014-2526-8 25420703

[bb12] Wang, Z., Wang, N., Han, S., Wang, D., Mo, S., Yu, L., Huang, H., Tsui, K., Shen, J. & Chen, J. (2013). *PLoS One*, **8**, e68566. 10.1371/journal.pone.0068566 PMC3702614 23861918

[bb13] Wolff, S., Grimwood, D., McKinnon, J., Turner, M., Jayatilaka, D. & Spackman, M. (2012). *CrystalExplorer*. University of Western Australia.

[bb14] Zaini, M. F., Arshad, S., Thanigaimani, K., Khalib, N. C., Zainuri, D. A., Abdullah, M. & Razak, I. A. (2019). *J. Mol. Struct.* **1195**, 606–619.

[bb15] Zaini, M. F., Razak, I. A., Khairul, W. M. & Arshad, S. (2018). *Acta Cryst.* E**74**, 1589–1594. 10.1107/S2056989018014329 PMC6218902 30443387

[bb16] Zaini, M. F., Razak, I. A., Khairul, W. M. & Arshad, S. (2019). *Acta Cryst.* E**75**, 685–689. 10.1107/S2056989019005243 PMC6505597 31110811

[bb17] Zainuri, D. A., Razak, I. A. & Arshad, S. (2018). *Acta Cryst.* E**74**, 1302–1308. 10.1107/S2056989018011131 PMC6127721 30225122

[bb18] Zhuang, C., Zhang, W., Sheng, C., Zhang, W., Xing, C. & Miao, Z. (2017). *Chem. Rev.* **117**, 7762–7810 10.1021/acs.chemrev.7b00020 PMC6131713 28488435

