# Supplementary material for: Structural, Hirshfeld and DFT studies of conjugated D–π–A carbazole chalcone crystal
Source: Acta Crystallogr E Crystallogr Commun. 2020 Feb 18;76(Pt 3):387–91. doi: 10.1107/S2056989020002054 (PMC7057368; doi:10.1107/S2056989020002054)
Supplement: Supplementary file 4 [file e-76-00387-sup3.docx]

**Supplementary Information (SI) of CPNC**

**SI** Comparison between the calculated optimized and X-ray geometrical parameters for the **CPNC** compound.

|  | **CPNC** | |
| --- | --- | --- |
|  | X-ray | DFT  B3LYP/6-311G++(d,p) |
| Bond length (Å) | | |
| O1—C21 | 1.215 (3) | 1.223 |
| O2—N2 | 1.227 (3) | 1.224 |
| O3—N2 | 1.222 (3) | 1.224 |
| N1—C1 | 1.404 (3) | 1.402 |
| N1—C13 | 1.425 (3) | 1.414 |
| N2—C25 | 1.472 (3) | 1.482 |
| C16—C19 | 1.471 (3) | 1.457 |
| C19—C20 | 1.320 (3) | 1.348 |
| C20—C21 | 1.485 (3) | 1.478 |
| C21—C22 | 1.507 (3) | 1.509 |
| Bond angle (°) | | |
| C1—N1—C13 | 126.3 (19) | 125.9 |
| O2—N2—O3 | 123.6 (2) | 124.8 |
| C25—N2—O3 | 118.5 (2) | 117.6 |
| O1—C21—C22 | 119.9 (2) | 119.2 |
| O1—C21—C20 | 121.9 (2) | 122.2 |
| C17—C16—C19 | 122.3 (2) | 123.5 |
| C16—C19—C20 | 126.4 (2) | 127.9 |
| C19—C20—C21 | 120.8 (2) | 120.3 |
| C20—C21—C22 | 118.2 (2) | 118.6 |
| C21—C22—C27 | 122.34 (19) | 123.1 |
| Torsion angle (°) | | |
| C1—N1—C13—C14 | 51.8 (4) | 53.2 |
| C15—C16—C19—C20 | 158.6 (3) | 178.8 |
| C19—C20—C21—O1 | −27.7 (4) | −4.4 |
| O1—C21—C22—C23 | −9.5 (4) | −18.6 |
| O1—C21—C22—C27 | 167.4 (3) | 159.7 |
| C16—C19—C20—C21 | −175.9 (2) | 178.7 |
| C19—C20—C21—C22 | 150.0 (2) | 176.7 |
| C20—C21—C22—C27 | −10.4 (3) | −21.3 |
| C20—C21—C22—C23 | 172.7 (2) | 160.3 |
